# Electrosurgical knife with the water‐jet function of tip‐type during endoscopic treatment injection

**DOI:** 10.1002/deo2.165

**Published:** 2022-09-20

**Authors:** Satoshi Ono, Yasuko Kurihara, Fumiya Hirose, Hajime Aoki, Kyohei Mejima, Shun Ito, Shun Osumi, Seika Hatori, Kazushi Fukagawa, Shosuke Hosaka, Miho Matsukawa, Ryosuke Kobayashi, Nobuyoshi Yamazaki, Mitsuhiro Fujishiro

**Affiliations:** ^1^ Department of Gastroenterology and Gastrointestinal Endoscopy Tokyo Metropolitan Geriatric Medical Center Tokyo Japan; ^2^ Department of Gastroenterology Chiba‐Nishi General Hospital Chiba Japan; ^3^ Department of Gastroenterology The University of Tokyo Hospital Tokyo Japan; ^4^ Department of Clinical Engineering Chiba‐Nishi General Hospital Chiba Japan; ^5^ Department of Surgery Chiba‐Nishi General Hospital Chiba Japan

**Keywords:** desktop experiment, electrosurgical knife, endoscopic submucosal dissection, injection, porcine model

## Abstract

**Objectives:**

This study aimed to objectively evaluate the water‐jet‐functioned electrosurgical knife injection performances in a desktop experiment.

**Methods:**

Five types of water‐jet‐functioned electrosurgical knives, including two injection styles of sheath‐type (A: DualKnife J, KD‐655L; B: FlushKnife, DK2620‐J‐B20S; C: Splash M‐Knife, DN‐D2718B; D: ISSEN, SN1650‐20) and tip‐type (E: ORISE ProKnife, M00519361) were evaluated. These knives were compared with an injection needle (Control: SuperGrip 25G) as a control. The injection speed under constant pressure and the injection efficiency for each knife against prepared porcine stomach mucosa were evaluated. The additional clear gel injections using an injection needle were observed using an indigo blue‐colored gel to evaluate the difference between the locations of water‐jet holes.

**Results:**

Four types of knives, except for A, showed significantly higher water‐jet speeds (A: 0.79 ± 0.03 g/20 s, B: 2.56 ± 0.05 g/20 s, C: 3.09 ± 0.06 g/20 s, D: 2.86 ± 0.05 g/20 s, and E: 1.79 ± 0.03 g/20 s) compared to that of the control (1.21 ± 0.03 g/20 s). Meanwhile, significantly higher efficacy of injection was found in the tip‐type water‐jet function knife, second to the injection needle (Control: 37.2% ± 35.5%, A: 20.9% ± 20.2%, B: 1.1% ± 2.2%, C: 6.2% ± 12.6%, D: 12.5% ± 15.6%, and E: 33.3% ± 32.2%). An additional injection experiment revealed that the injection with a piercing tip into the gel could achieve sufficient additional injection inside the stacked clear gel.

**Conclusions:**

The tip‐type water‐jet function electrosurgical knife is preferable for effective submucosal injection during endoscopic treatments.

## INTRODUCTION

Nowadays, endoscopic treatments have been established as reliable options for various gastrointestinal diseases. Endoscopic submucosal dissection has been accepted as the treatment of choice for gastrointestinal neoplasms with a low possibility of lymph node metastases.^1^, ^2^, ^3^ Additionally, laparoscopy and endoscopy cooperative surgery, non‐exposed endoscopic wall‐inversion surgery, and submucosal tunneling endoscopic resection demonstrate the novel possibility of endoscopic treatment for a submucosal tumor in the gastrointestinal tract.^4^, ^5^, ^6^ Peroral endoscopic myotomy enabled the treatment of achalasia and spastic esophageal disorders that do not respond to medical therapies.^7^, ^8^

All these procedures consist of the mucosal incision and the submucosal dissection using electrosurgical knives and injection needles. During these steps, repetitive appropriate submucosal injections are mandatory to safely conduct procedures. However, frequent electrosurgical knife and injection needle exchanges are troublesome and time‐loosing. Recently, various electrosurgical knives with water‐jet functions are available worldwide. They enable additional injections to keep an appropriate fluid cushion during the submucosal dissection. However, their injection performances using water‐jet functions have not been objectively evaluated thus far.

Therefore, this study aimed to objectively evaluate the injection performances of electrosurgical knives with water‐jet functions in a desktop experiment.

## MATERIALS AND METHODS

**FIGURE 1:**
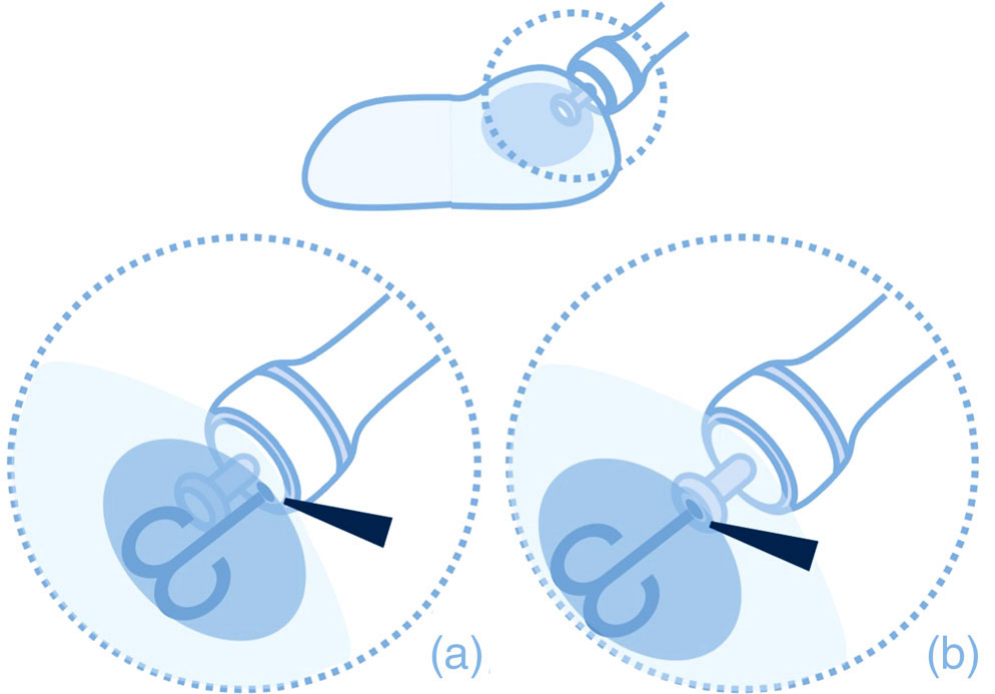
The scheme of the sheath‐type and the tip‐type knives. Arrowheads indicate the hole of the water jet. (a) The sheath‐type knife. (b) The tip‐type knife

Five types of electrosurgical knives (A: DualKnife J, KD‐655L; B: FlushKnife, DK2620‐J‐B20S; C: Splash M‐Knife, DN‐D2718B; D: ISSEN, SN1650‐20, E: ORISE ProKnife, M00519361) with water‐jet functions from five manufacturers (Olympus Corp., Tokyo, Japan; Fujifilm Corp., Tokyo, Japan; Hoya Corp., Tokyo, Japan; Kaneka Medix Corp., Osaka, Japan; and Boston Scientific Corp., Massachusetts, USA) were evaluated. These knives were compared with one type of injection needle (SuperGrip 25G; TOP Corp., Tokyo, Japan). Their characteristics, including length, injection styles, tip‐type, or sheath‐type, are summarized in Table 1. Injection styles of water shooting through a small hole of the sheath and through a small hole of the metal tip were defined as sheath‐type and tip‐type, respectively (Figure 1). Among them, E is the only knife with a water‐jet function in tip‐type.

**TABLE 1:** The characteristics of the electrosurgical knives with the tip‐type water‐jet function

	**Device**	**Injection style**	**Length of tip (mm)**	**Maximal diameter of sheath (mm)**	**Length of sheath (mm)**
Control	SuperGrip25G	Tip‐type	3.0	2.7	1600
A	DualKnife J	Sheath‐type	2.0	2.7	1650
B	FlushKnife	Sheath‐type	2.0	2.7	2000
C	Splash M‐Knife	Sheath‐type	2.0	2.7	1800
D	ISSEN	Sheath‐type	2.0	2.75	1650
E	ORISE Proknife	Tip‐type	2.0	2.7	2300

### Water‐jet speed under constant pressure

**FIGURE 2:**
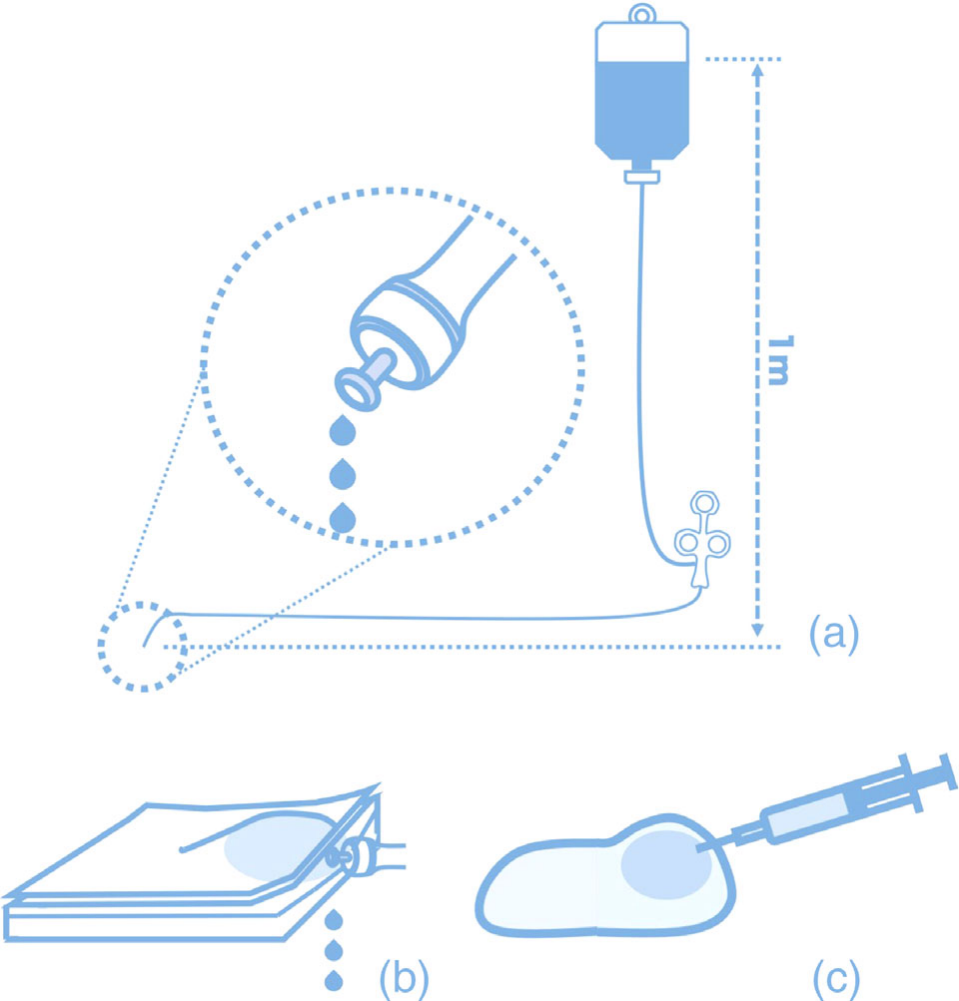
The scheme of measurements. (a) The injection speed under constant pressure. (b) The efficiency of injection. (c) The experiment of additional injections

The knife laid upon a flat floor was connected by infusion route to a bottle of normal saline that is located 1 m above as shown in Figure 2a. The weight of water droplets from the tip of the knives in 20 s was evaluated by the electro weighing scale (ACS200; AS ONE Corp., Osaka, Japan). The measurements were repeated 10 times for each knife, and the mean value of the water droplets’ weight per 20 s was calculated.

### Efficiency of injection

Porcine stomachs of the lower part that are cut into rectangles were prepared for direct submucosal injection evaluation. Piercing the tip of the knife to the submucosa approximately 2–3 mm until its metal tip is hidden, 2 ml or 2 g of normal saline was manually injected into the submucosa within 5 s as shown in Figure 2b. The injection speed was controlled similarly to the speed in the clinical daily practice. The total amount of injected agents was evaluated by comparing the weight before and after the injection. The ratio of the gained weight to 2 g of the injected agent was defined as the injection efficacy. The injection conditions of the porcine stomach are more varied than the human stomach; thus, the measurements were repeated 10 times and the larger five values for each knife were adopted. The mean value of the ratio of the injected agent to 2 g of the total amount was calculated.

### Difference between the locations of the hole of the water jet

To visualize the differences between the locations of the hole of the water jet, an experiment of additional injection using gel and needles were conducted. The additional injection was simulated using clear gel (COMF Pro Lubricating jelly: F‐three Corp., Nagoya, Japan) and indigo blue‐colored gel, including a very small amount of indigo carmine. These materials were used because of their transparency which is helpful in visually understanding the distribution of additional blue gels although the electrosurgical knives could not be used due to their high viscosity. A piercing of 2 mm of the tip of the 25G needle was made into the clear gel stacked on the water repellent sheet, and 1 ml of additional blue‐colored gel was manually injected as shown in Figure 2c. On the other hand, a control experiment was made by contacting the tip of a 25G needle on the surface of clear gel stacked, and 1 ml of additional blue‐colored gel was injected.

The calculations of the *t*‐test were done using EXCEL software (Microsoft Corp., Seattle, Washington, U). A *p*‐value <0.05 was considered statistically significant.

## RESULTS

**FIGURE 3:**
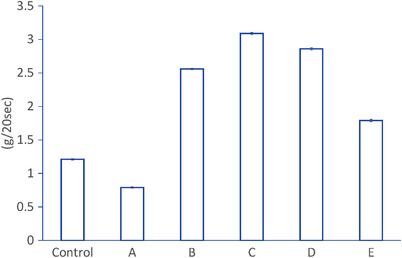
The mean values and standard errors of the injection speed under constant pressure

The results of the water‐jet speed under the constant pressure of each knife are shown in Figure 3. Compared to the water‐jet speed of the control (1.21 ± 0.03 g/20 s), four types of knives, except for A, showed a higher water‐jet speed than injection needles (A: 0.79 ± 0.03 g/20 s, B: 2.56 ± 0.05 g/20 s, C: 3.09 ± 0.06 g/20 s, D: 2.86 ± 0.05 g/20 s, and E: 1.79 ± 0.03 g/20 s). The *p*‐values of the student *t*‐test for combinations of knives and an injection needle were all <0.05.

**FIGURE 4:**
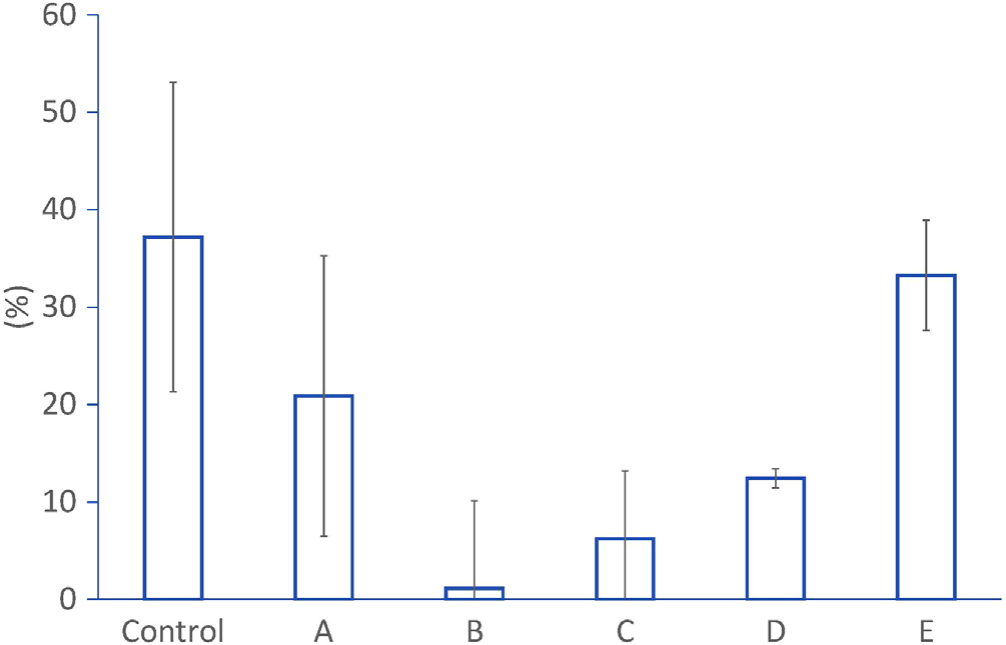
The mean values and standard errors of the efficiency of injection

Meanwhile, the injection efficacy results revealed that the higher water‐jet speed did not necessarily correspond to the injection efficacy as shown in Figure 4. The knife with the tip‐type water‐jet function (E: 33.3 ± 32.2%) was with higher injection efficacy second to the injection needle (37.2% ± 35.5%). It was also significantly higher than the other knives of the sheath‐type water‐jet function (A: 20.9% ± 20.2%, B: 1.1% ± 2.2%, C: 6.2% ± 12.6%, and D: 12.5% ± 15.6%). The p‐values of the student *t*‐test for combinations of the knife with the tip‐type water‐jet function and the other knives with the sheath‐type water‐jet function were all <0.05.

**FIGURE 5:**
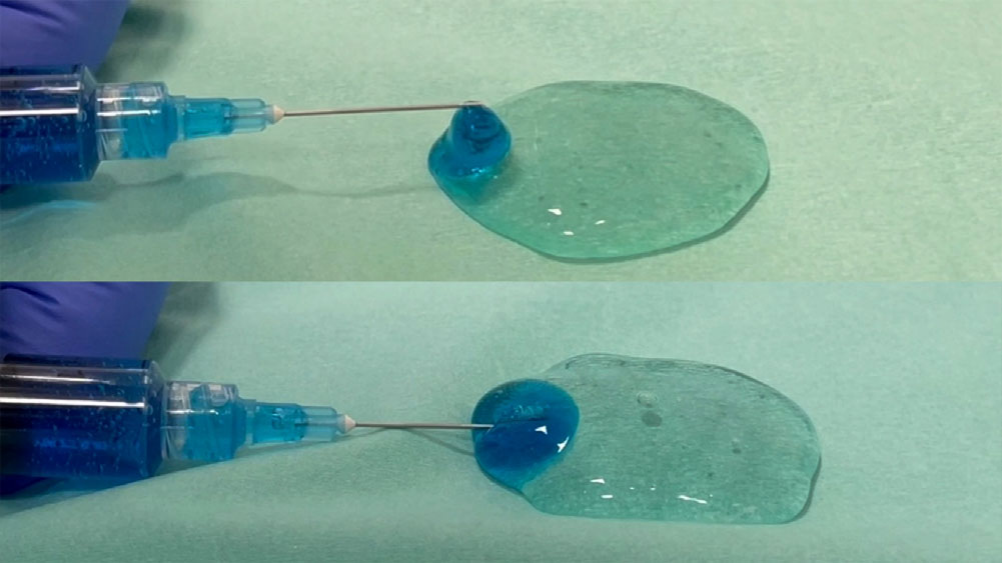
The images of additional injection by the needles in two locations. Upper image: The tip of the needle was kept on the surface of the gel. Lower image: The tip of the needled was kept inside of the gel

The results of the experiment concerning the differences between the locations of the hole of the water jet are shown in Figure 5 and Video S1. The injection with a piercing tip into the stacked gel could achieve sufficient additional injection into the inside of the stacked clear gel although the injection with contacting tip on the stacked gel surface just deposited additional blue‐colored gel on the stacked gel surface.

## DISCUSSION

Electrosurgical knives with water‐jet functions have been accepted as mandatory to conduct safe and smooth endoscopic procedures. The performances of these knives vary by combinations of injection agents although their differences have not been objectively evaluated thus far. This is the first study concerning the objective evaluation of the performances of the electrosurgical knives with the sheath‐type and the tip‐type water‐jet functions. It revealed that the electrosurgical knife with the tip‐type injection could have higher efficacy for additional injections during procedures.

From the standpoint of hydrodynamics, the fluid dynamics through a thin tube can be represented by the Hagen–Poiseuille equation as shown below^9^:

(1) $$delta-p=8\;\mu LQ/\pi R^{4}$$

where *delta‐p* is the pressure difference between the two ends, μ is the dynamic viscosity, *L* is the length, *Q* is the volumetric flow rate and *R* is the pipe radius.

Among these factors, the dynamic viscosity depends on the injection agent materials although other factors depend upon the structural characteristics of the knives. Meanwhile, hyaluronate acid preparations are widely used injection agents to keep appropriate submucosal cushions during endoscopic procedures although their dynamic viscosity is high.^10^, ^11^ Therefore, hyaluronate acid preparations are preferred for knives with higher water‐jet speeds.

On the other hand, higher injection efficacy is required from the standpoint of the economic costs of hyaluronic acid preparations. In this study, the knife with the tip‐type water‐jet function demonstrated a higher injection efficacy. Therefore, hyaluronic acid preparation is preferred for the knife with tip‐type water‐jet function although the knives with sheath‐type water‐jet function are acceptable with normal saline.

In this study, the injection efficacies of the three types of knives with sheath‐type injection showed less than half of that of the knife with tip‐type injection. Based upon the experience of use in the clinical daily practice, such differences were not expected. This discrepancy might be caused by the experimental design using a porcine stomach cut into rectangles. As mentioned above, the injection conditions of the porcine stomach are more varied than in the human stomach. Additionally, an excessive mucosal incision should be avoided in the actual endoscopic procedures to prevent the outflow of the injected agents. Therefore, the pocket creation method or the tunneling method, as the minimal mucosal incision, might reduce the loss of injection agents.^12^, ^13^, ^14^ Under such procedures, the injection performance of each knife might be improved.

Undoubtedly, one of the major limitations of this study is its study design as an experimental model using the porcine stomach mucosa. It is known that the porcine stomach submucosa is stiffer than the human stomach submucosa. Therefore, the results obtained in this study are not necessarily reflective of the actual situation of endoscopic procedures in the human gastrointestinal tract. Additionally, the injection pressure is not always tightly controlled because it is a manual injection. Moreover, the structural characteristics of the knives could not be evaluated enough because some of this data is not published. However, the tip‐type water‐jet function might be preferable considering the injection efficacy results and the experiment of additional injections, especially for higher dynamic viscosity agents, including hyaluronic acid preparations. Certainly, the water‐jet function of the tip‐type is inferior to an injection needle although it can reduce the exchanges of electrosurgical knives and injection needles.

In conclusion, this study on the objective evaluations of the water‐jet function of the electrosurgical knives revealed the effective injection of the knife with the tip‐type water‐jet function in experimental models. Such types of electrosurgical knives might be preferable for endoscopic procedures although further evaluations in clinical trials are mandatory.

## CONFLICT OF INTEREST

MF received a research grant from Fujifilm Corporation, HOYA Corporation, and Olympus Corporation and a lecture honoraria from Fujifilm Corporation and Olympus Corporation outside the submitted work.

## FUNDING INFORMATION

None.

## Supporting information


**VIDEO S1** The video comparison of the experiment of additional injections.
